# Elucidating Early Radiation-Induced Cardiotoxicity Markers in Preclinical Genetic Models Through Advanced Machine Learning and Cardiac MRI

**DOI:** 10.3390/jimaging10120308

**Published:** 2024-12-01

**Authors:** Dayeong An, El-Sayed Ibrahim

**Affiliations:** 1 Department of Biomedical Engineering, Medical College of Wisconsin, Milwaukee, WI 53226, USA; dan@mcw.edu; 2 Department of Radiology, Medical College of Wisconsin, Milwaukee, WI 53226, USA

**Keywords:** radiation-induced heart dysfunction, machine learning in medical imaging, cardiac magnetic resonance imaging, myocardial strain analysis, cardiotoxicity

## Abstract

Radiation therapy (RT) is widely used to treat thoracic cancers but carries a risk of radiation-induced heart disease (RIHD). This study aimed to detect early markers of RIHD using machine learning (ML) techniques and cardiac MRI in a rat model. SS.BN3 consomic rats, which have a more subtle RIHD phenotype compared to Dahl salt-sensitive (SS) rats, were treated with localized cardiac RT or sham at 10 weeks of age. Cardiac MRI was performed 8 and 10 weeks post-treatment to assess global and regional cardiac function. ML algorithms were applied to differentiate sham-treated and irradiated rats based on early changes in myocardial function. Despite normal global left ventricular ejection fraction in both groups, strain analysis showed significant reductions in the anteroseptal and anterolateral segments of irradiated rats. Gradient boosting achieved an F1 score of 0.94 and an ROC value of 0.95, while random forest showed an accuracy of 88%. These findings suggest that ML, combined with cardiac MRI, can effectively detect early preclinical changes in RIHD, particularly alterations in regional myocardial contractility, highlighting the potential of these techniques for early detection and monitoring of radiation-induced cardiac dysfunction.

## 1. Introduction

Radiation therapy (RT) has a vital role in the definitive treatment of various cancers of the thorax, including breast, lung, and esophageal cancers, as well as in young patients with lymphomas or other chest tumors. A significant clinical issue for many patients treated for thoracic cancers is radiation-induced heart dysfunction (RIHD), often resulting from incidental heart radiation as part of their cancer treatment [1,2,3]. A critical gap in the current understanding of RIHD is the lack of early biomarkers. This deficiency hampers the ability to detect and intervene before significant cardiac dysfunction occurs, underscoring the urgent need for research focused on identifying sensitive and specific markers that can predict RIHD at an early stage [4,5].

We have developed a pre-clinical RIHD model that demonstrates that genetic variants in rat chromosome 3 can change the heart sensitivity to RIHD. Specifically, chromosome 3 genetic variants derived from the inbred Brown Norway (BN) strain led to more resistance than chromosome 3 variants from the salt-sensitive (SS) strain. This was demonstrated by comparing SS rats with chromosome 3 from the BN strain (termed SS.BN3 consomic rats) vs. SS rats [6]. In contrast, the increased sensitivity to RT in SS rats is manifested through heightened pleural and pericardial effusions, echocardiogram parameters indicate deteriorated left ventricular (LV) function, and histology demonstrating more myocardial necrosis [6,7].

Currently, cardiac function assessment primarily relies on measuring LV ejection fraction (EF) [8]. However, EF diagnostic and prognostic capabilities are limited, as they may not accurately represent regional cardiac dysfunction [9]. Consequently, there is a need to identify sensitive markers for the early detection of RT-induced cardiotoxicity in patients receiving heart radiation [10]. Magnetic resonance imaging (MRI) is the reference standard for cardiac function evaluation [11,12,13,14]. Myocardial strain imaging by MRI is a sensitive measure for the early detection of cardiac dysfunction and identifying patients at risk of developing heart failure (HF) [9,15,16,17,18,19]. Yet, MRI strain imaging in studying RT-induced cardiotoxicity is still underexplored [20].

Machine learning (ML) has emerged as a powerful tool in medical imaging and diagnostics [21,22]. ML algorithms can process large volumes of imaging data to detect subtle changes in cardiac structure and function, enabling the earlier and more accurate diagnosis of conditions like RIHD. By applying ML techniques to MRI strain imaging, the sensitivity and specificity of detecting radiation-induced cardiotoxicity at a preclinical stage can be enhanced. This integration not only facilitates the identification of patients at risk before significant EF changes occur but also addresses challenges related to data complexity and interpretation. Leveraging ML’s capabilities thus holds promise for advancing early detection strategies and improving clinical outcomes for patients undergoing thoracic RT.

This study emphasizes the application of ML techniques to detect and analyze these effects, particularly in preclinical stages of RIHD. The integration of ML with advanced MRI techniques represents a novel approach to overcoming challenges in widespread clinical adoption due to complexity and interpretation difficulties. By applying sophisticated ML algorithms, we aim to simplify and enhance the detection of early cardiac changes induced by radiation, offering a more accessible pathway for identifying patients at risk.

This study’s use of the SS.BN3 rat model, with its subtler phenotype than the more radiation-sensitive SS Dahl rat, enables the detection of preclinical changes before significant EF alterations occur, indicating the potential of ML to uncover associations within imaging data that traditional methods might not reveal.

## 2. Materials and Methods

### 2.1. Animal Model

The SS.BN3 model was used in this study. The SS.BN3 consomic rats are derived from selective breeding, where they are genetically identical to the SS rats, except for chromosome 3, which is inherited from the BN strain, as explained in our earlier work [7]. Briefly, we have identified the parental Dahl SS rat strain to be a highly sensitized model of RT-induced cardiotoxicity. Alternatively, using rat chromosome 3 from the resistant BN rat strain onto the SS background produced the SS.BN3 rat model, which has significantly attenuated radiation-induced cardiotoxicity, as previously demonstrated [7].

### 2.2. Study Design

The study was approved by our institutional animal care and use committee (IACUC). Adult female SS.BN3 rats, aged 10 weeks, received localized whole-heart RT and were imaged with MRI at 8 weeks (*n* = 5) and 10 weeks (*n* = 9) post-RT, as previously described [4,7]. Another group of SS.BN3 rats (*n* = 6) received sham treatment at 10 weeks of age and were imaged at 8 weeks post-sham to serve as non-irradiated controls. All rats were scanned on a 9.4T Bruker Biospec MRI scanner (Bruker, Billerica, MA, USA) with 30 cm bore diameter and equipped with a 4-element surface coil.

### 2.3. Heart Irradiation

The SS.BN3 rats were subjected to localized heart irradiation using the high-precision image-guided X-RAD SmART irradiator (Precision X-Ray, North Branford, CT, USA). The output of the SmART was regularly checked using a calibrated ionization chamber. The rats were anesthetized by 3% isoflurane/room temperature air inhalation for the duration of each treatment, where they were prone positioned. A circular 1.5 cm diameter collimator was used that encompassed the whole heart. The central axis of the beam was aligned with the heart’s center. A radiation dose to the isocenter was administered at 24 Gy × 1 fraction, with equally weighted anterior–posterior and two lateral beams (1:1:1, 225 kVp, 13 mA, 0.32 mm Cu, 2.69 Gy/min). Sham SS.BN3 rats were subjected to sham irradiation.

### 2.4. Animal Care and Monitoring

All rats were housed in temperature-controlled rooms with a 12 h light/dark cycle and had free access to food and water. Veterinary staff routinely monitored the health and welfare of all rats, ensuring adherence to the highest standards of animal care.

### 2.5. MRI Scans

MRI scans were conducted, which included both long-axis (LAX) and short-axis (SAX) cine and tagged images. A total of 2400 images were acquired using fast low angle short (FLASH) pulse sequences with both cardiac respiratory gating. The imaging parameters were optimized for all pulse sequences, as previously described [23]. The cine sequence imaging parameters were as follows: repetition time (TR) = 7 {ms, echo time (TE) = 2.1 ms, flip angle = 15°, matrix = 176 × 176, field of view (FOV) = 40 × 40 ${\mathrm{mm}}^{2}$, slice thickness = 1 mm, acquisition bandwidth = 526 Hz/pixel, # averages = 2, # cardiac phases = 20, and scan time ~2 min per slice. The tagging sequence imaging parameters were as follows: TR = 7 ms, TE = 2.5 ms, flip angle = 15°, matrix = 256 × 256, FOV = 40 × 40 ${\mathrm{mm}}^{2}$, slice thickness = 1 mm, acquisition bandwidth = 375 Hz/pixel, # averages = 3, # cardiac phases = 20, and scan time = 4–5 min per slice.

### 2.6. Image Analysis

The cine images (20 per slice) were analyzed using Circle cvi42 software (Circle Cardiovascular Imaging, Calgary, AB, Canada) to measure ventricular EF and myocardial mass. The tagged images (20 per slice) were analyzed using the SinMod method [24] (InTag software, Lyon, France) to measure myocardial circumferential strain (Ecc), radial strain (Err), longitudinal strain (Ell), rotation angle, SAX tissue motion, and LAX tissue motion. In accordance with the American Heart Association’s (AHA) guidelines [25], each of these parameters was analyzed based on the AHA 6-segment model, which divides the heart into three slices (basal, mid-ventricular, and apical) with a total of sixteen segments per heart. This segmentation ensures a standardized approach to assess and interpret regional myocardial function. In addition, regional cardiac function parameters were measured at different ventricular regions.

### 2.7. Feature Selection

A total of 24 features were available for the study. These were Ecc, Err, rotation angle, and SAX tissue motion based on the AHA 6-segment per slice model. The dataset was split into training and testing sets at a 70% to 30% ratio. To identify the most informative features for classification between sham and irradiated rats, various feature selection techniques were employed. The recursive feature elimination (RFE) technique was utilized, which systematically removes the least important features and retrains the model until the desired number of features is reached. For the logistic regression (LR) [26] model, the estimator was initialized with a maximum iteration of 10,000 to ensure convergence. Subsequently, RFE was employed to select the most significant feature for this model. For the support vector machine (SVM) [27] model, a linear kernel was used, as RFE requires a linear approach for SVMs. Both models were subjected to RFE to rank the features based on their significance. In order to complement the feature selection approach, the random forest (RF) classifier [28] was also explored. After being trained on the scaled training data, the feature importance provided by this model were evaluated. The most significant features were extracted and listed based on their importance scores, providing a hierarchical understanding of each feature’s significance in the dataset. Spearman correlation coefficients identified highly correlated features, setting the threshold at 0.7 and −0.7. The features chosen from these rankings were then used for further training and evaluation.

### 2.8. Machine Learning

Five ML algorithms were employed as follows: LR [26], SVM [27], RF [28], gradient boosting (GB) [29], and multi-layer perceptron neural networks (NN) [30]. Firstly, LR is a statistical method used for predicting the probability of a certain class or event. LR estimates the relationship between the dependent and independent variables using a logistic function. Secondly, the SVM is a supervised learning algorithm that finds a hyperplane in an N-dimensional space, distinctly classifying data points. SVM is robust in handling non-linear data patterns and operates effectively in high-dimensional spaces. The third algorithm, RF, is an ensemble learning method that employs multiple decision trees during training and outputs the mode of the classes for prediction. It inherently assesses feature importance. Fourthly, GB method builds an additive model iteratively, optimizing arbitrary differentiable loss functions. It adds weak learners in each iteration to minimize the residual error. Lastly, the NN are feed forward artificial neural networks composed of interconnected nodes or neurons. They capture intricate patterns and relationships in data, making them apt for complex classification tasks. All selected features from the training dataset were standardized to z-scores using the sample means and standard deviations, ensuring unbiased algorithm performance due to disparate feature scales.

### 2.9. Evaluation Metrics

The classification performance [31,32] of the different machine learning models in the testing stage was compared based on the scores of accuracy (measures the fraction of predictions that the model determined correctly), F1 scores (the harmonic mean of precision and recall, providing a balance between the two metrics), specificity (measures the fraction of actual negatives that were correctly identified), sensitivity (measures the fraction of actual positives that were correctly identified), and area under the receiver operating characteristic (ROC) curve (measures the true positive rate against the false positive rate, providing the model’s discriminative ability).

### 2.10. Statistical Analysis

Student’s *t*-test was conducted to identify significant measurement differences between different rat groups and imaging timepoints. For comparisons involving more than two groups, ANOVA [33] was utilized, followed by post hoc tests where appropriate. In cases where data did not meet the assumptions of normality, non-parametric equivalents, Mann–Whitney U [34] or Kruskal–Wallis [35] tests, were employed. The data were expressed as mean ± standard deviation (SD).

## 3. Results

### 3.1. Normal EF Is Maintained Post-RT

**Figure 1:**
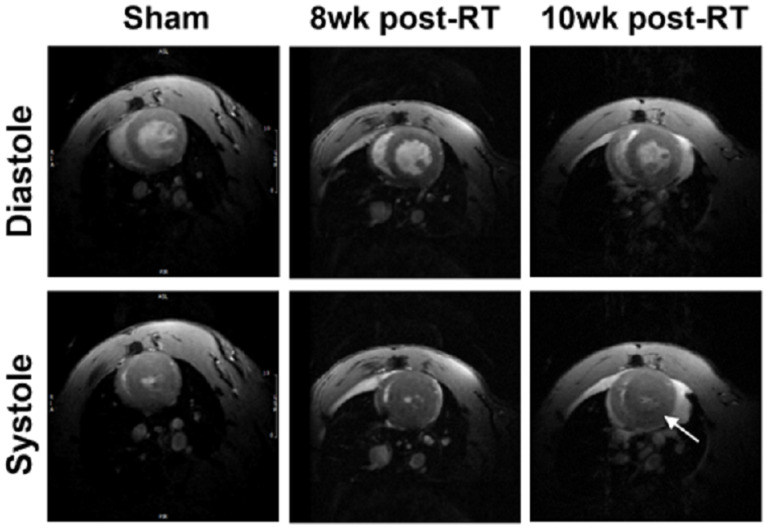
Ventricular remodeling post-RT to preserve global cardiac function. Mid-ventricular short axis cine images showing end-diastolic and end-systolic images in sham, 8 weeks post-RT, and 10 weeks post-RT in SS.BN3 rats. The images show preserved cardiac function post-RT, along with concentric hypertrophy (arrow).

Figure 1 shows both end-diastolic (ED) and end-systolic (ES) cine images of the sham and RT rats. Global cardiac function, as assessed by EF, was normal in all rats, with increased EF and myocardial mass in the RT, compared to sham measurement. The increased level of LV myocardial mass was stable at 8 weeks and 10 weeks post-RT. LV EF was 72 ± 1.2%, 80 ± 1.7% (*p* = 0.003), and 76 ± 1.1% (*p* = 0.013) in the sham, 8 weeks post-RT and 10 weeks post-RT, respectively. LV mass was 0.36 ± 0.02 g, 0.46 ± 0.03 g (*p* = 0.02), and 0.44 ± 0.02 g (*p* = 0.003) in the sham, 8 weeks post-RT, and 10 weeks post-RT, respectively.

### 3.2. Myocardial Strain and Tissue Displacement Differentiate Cardiac Response to RT

**Figure 2:**
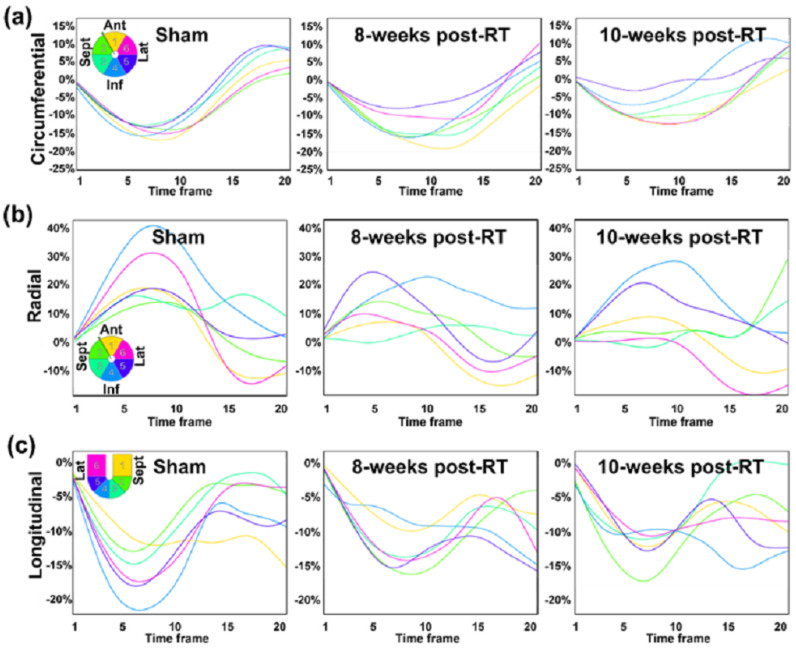
Contractility pattern changes post-RT. Segmental (**a**) circumferential, (**b**) radial, and (**c**) longitudinal strain curves throughout the whole cardiac cycle (20 timeframes starting after the R-wave of the ECG signal) in SS.BN3 sham, 8 weeks post-RT, and 10 weeks post-RT rats. Myocardial segmental color code is shown on the left panel in each row (short-axis slices for circumferential and radial strain and long-axis slice for longitudinal strain), where Ant, Inf, Sept, and Lat show the anterior, inferior, septal, and lateral segments, respectively. Note reduced peak strain post-RT. Note more heterogeneity (mechanical dyssynchrony) between strain curves from different heart segments at 10 weeks post-RT.

**Figure 3:**
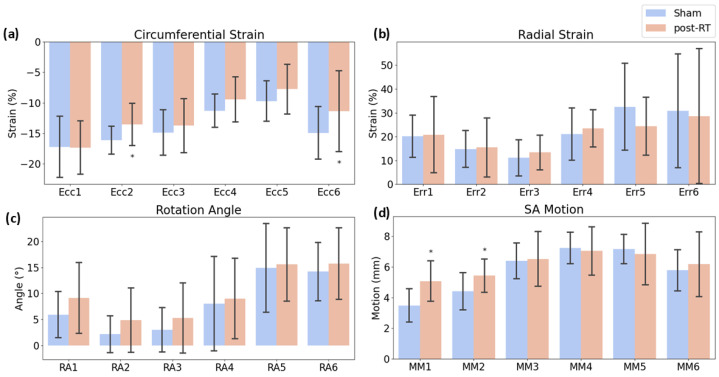
The bar plots illustrate the differences across four key metrics: (**a**) circumferential strain, (**b**) radial strain, (**c**) rotation angle, and (**d**) short-axis (SAX) motion. Data are represented for the six myocardial sectors: anterior, anteroseptal, inferoseptal, inferior, inferolateral, and anterolateral. Error bars show SEM. Asterisk (*) indicates statistically significant (*p* < 0.05) differences between sham and post-RT.

**Figure 4:**
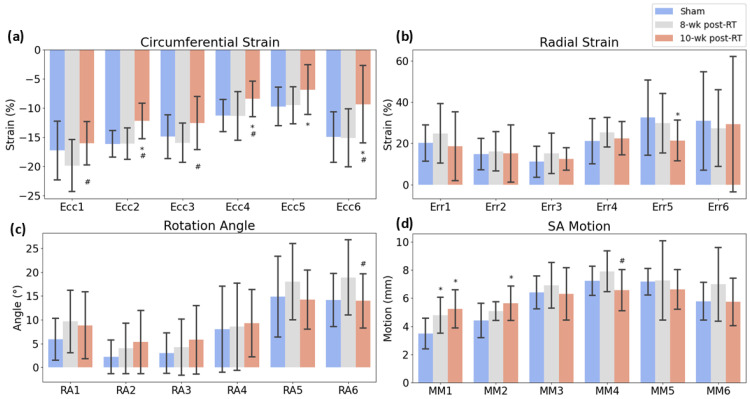
Thebar plots illustrate the differences across four key metrics: (**a**) circumferential strain, (**b**) radial strain, (**c**) rotation angle, and (**d**) short-axis (SAX) motion. Data are represented for the six myocardial sectors: anterior, anteroseptal, inferoseptal, inferior, inferolateral, and anterolateral. Error bars show SEM. Asterisk (*) indicates statistically significant (*p* < 0.05) differences between sham and 8 weeks post-RT or 10 weeks post-RT. Hash (#) indicates statistically significant differences between 8 weeks post-RT and 10 weeks post-RT.

Figure 2 shows representative strain curves in both sham and RT SS.BN3 rats at different timepoints. Despite normal global function, strain measurements showed reduced (absolute) values in the radiation-treated compared to sham-treated rats. Table 1 shows strain and displacement measurements at different timepoints. Strain measurements were analyzed using ANOVA, revealing significant reductions in specific segments. Figure 3 contrasts the sham and irradiated (both at 8 weeks and 10 weeks post-RT) rats using the AHA model. For the non-normally distributed data, Mann–Whitney U tests were applied, particularly for the strain measurements in the anteroseptal and anterolateral segments manifested statistically significant reductions when compared to sham. For radial strain, some segments showed an increment post-RT, while others displayed a reduction. As for the rotation angle, although there was not a statistically significant difference across the segments, all segments experienced an increase relative to the sham rats. In SAX motion, the anterior and anteroseptal segments notably rose in comparison to the sham. Figure 4 offers a comparison between the sham, 8 weeks post-RT, and 10 weeks post-RT. In the context of circumferential strain, the anteroseptal, inferior, inferolateral, and anterolateral segments showed significant differences between the sham and 10 weeks post-RT. Moreover, with the exception of the inferolateral segment, all other segments exhibited substantial differences between 8 weeks post-RT and 10 weeks post-RT. Concerning radial strain, only the inferolateral segment presented a notable difference between the sham and 10 weeks post-RT. For the rotation angle, only the anterolateral segment demonstrated a significant discrepancy between 8 weeks and 10 weeks post-RT. In the SAX motion, the anterior segment revealed significant differences when comparing sham vs. both 8 weeks post-RT and 10 weeks post-RT. Additionally, the anteroseptal segment was significantly different when comparing the sham and 10 weeks post-RT, while the inferior segment showed a significant variation between 8 weeks and 10 weeks post-RT.

### 3.3. Performance Analysis of Machine Learning Models for Sham vs. Irradiated SS.BN3 Rats Classification

**Figure 5:**
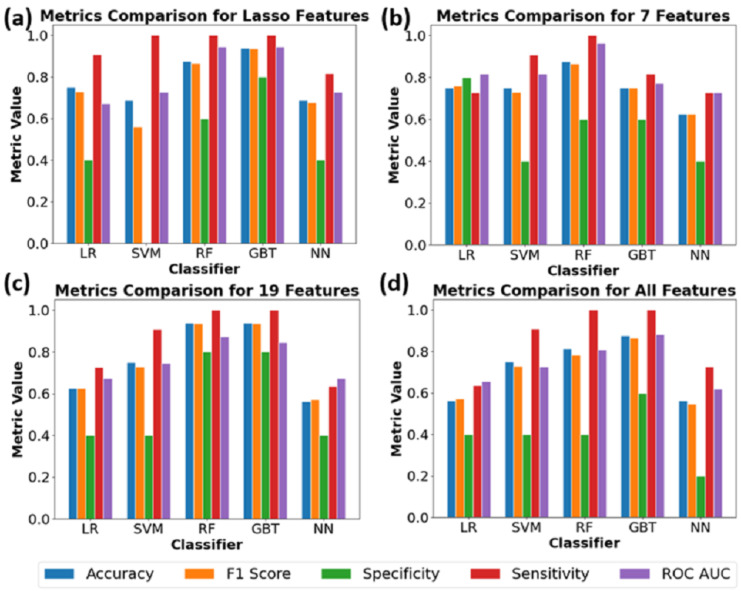
Comparison of performance metrics across different classifiers for various feature sets to differentiate sham vs. irradiated SS.BN3 rats. Bars represent metrics (accuracy, F1 score, specificity, sensitivity, ROC AUC) for feature sets (**a**) Lasso, (**b**) selected 7 features, (**c**) selected 19 features, and (**d**) all features.

**Figure 6:**
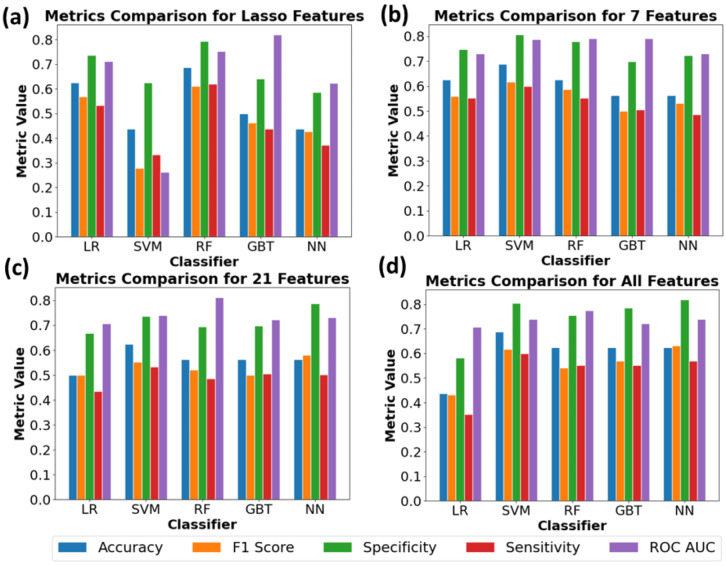
Comparison of performance metrics across different classifiers for various feature sets to differentiate sham vs. 8 weeks post-RT vs. 10 weeks post-RT. Bars represent metrics (accuracy, F1 score, specificity, sensitivity, ROC AUC) for feature sets (**a**) Lasso, (**b**) selected 7 features, (**c**) selected 21 features, and (**d**) all features.

Given the differences and complex patterns identified between sham and RT treatments, there emerges a compelling necessity for employing advanced analytical methods to accurately classify and predict cardiac responses. The classification performance of various ML algorithms in distinguishing sham vs. irradiated rats (including 8 weeks post-RT and 10 weeks post-RT) in Figure 5, as well as in distinguishing sham vs. 8 weeks post-RT vs. 10 weeks post-RT in Figure 6, were evaluated using different feature selection methods.

Utilizing the Lasso Features for sham vs. irradiated classification (Figure 5), the GB algorithm exhibited superior results with the highest accuracy of F1 score at 0.94, and a remarkable ROC value of 0.95. The RF algorithm also demonstrated promising outcomes with an accuracy of 0.88 and an ROC score of 1.00 but struggles with specificity. When evaluating the seveb selected features, RF stood out with a peak accuracy of 0.88 and perfect sensitivity. Both LR and SVM, presented an ROC score of 0.82. Delving into the 19 selected features, both GB and RF outperformed with a leading accuracy of 0.88 and an unmatched sensitivity score of 1.00. SVM also displayed a notable sensitivity of 0.91. Considering the model trained on all features, GB consistently showed superior results with an accuracy of 0.88, a sensitivity of 1.00, and an ROC score of 0.86. The RF followed closely with commendable scores across the board. Feature importance analysis from the RF and GB models identified key variables contributing to the classification, providing insights into the factors influencing model predictions. In essence, GB and RF consistently emerged as the most reliable algorithms for the classification task across various feature selections.

When distinguishing sham vs. 8 weeks post-RT vs. 10 weeks post-RT (Figure 6) with the Lasso features, the RF and LR models performed comparably in terms of accuracy, each achieving a score of 0.62. The RF model showed a promising ROC value of 0.79, while the GB model surpassed other algorithms with an ROC of 0.83. When utilizing the seven selected features, the SVM model demonstrated an improved accuracy of 0.69, alongside RF and GB models, both of which exhibited strong ROC scores, at 0.79 and 0.80, respectively. For the 21 selected features, the accuracy remained consistent across the LR, SVM, and RF models, all being around 0.62. GB’s ROC score reached 0.76. Notably, when all features were considered, the RF model displayed an accuracy of 0.75 and a notable ROC value of 0.84.

## 4. Discussion

This study into the early markers of RIHD through advanced ML techniques and cardiac MRI reveals critical insights into the effects of RT on cardiac function. Despite the global cardiac function as measured by the EF remaining within normal ranges post-RT, our study identified significant alterations in myocardial strain and tissue displacement. These findings suggest that EF alone may not suffice to detect early changes induced by RT and the importance of more sensitive and specific diagnostic tools.

The application of myocardial strain analysis, particularly in anteroseptal and anterolateral segments, highlighted the potential of advanced imaging techniques to reveal early cardiac damage. These strain measurements, significantly reduced in irradiated rats, provide evidence of regional cardiac dysfunction that precedes detectable changes in global cardiac metrics such as EF. The understanding of cardiac response to RT, facilitated by imaging analyses, underscores the complexity of RIHD and the need for innovative approaches to its detection.

The performance of ML models, particularly the RF and GB algorithms, have shown exceptional accuracy and sensitivity. Their effective discrimination between sham and irradiated SS.BN3 rats not only offers the feasibility of ML approaches in enhancing diagnostic precision, but also hints at their capability to inform patient monitoring and treatment strategies. The success of these algorithms in classifying sham and irradiated groups and their temporal changes further emphasizes ML’s role in advancing the understanding of RIHD progression. Although RF and GB models provide higher accuracy, we interpreted their results by analyzing feature importance scores, which highlighted the key variables contributing to the classification outcomes.

Limitations of the current study include the use of a limited number of studied animals. This small sample size raises the potential risk of overfitting in our machine learning models, where algorithms may perform well on training data but may not generalize effectively to new data. Despite applying methods to reduce overfitting, the limited number of experimental rats may affect the robustness and generalizability of our findings. However, the results from this study showed differences in the spatial distribution and temporal progression of the contractility patterns between sham-treated and irradiated SS.BN3 rats. Therefore, further studies with larger numbers of animals and more study timepoints would be beneficial. Such studies will also allow for investigating radiation effects on different heart sub-structures and studying the effects of other genetic variations on RT-induced cardiotoxicity, with the overall goal of identifying risk factors of RT-induced cardiotoxicity to allow for optimal treatment planning and post-RT care that enhance the toxicity profile and improve treatment outcomes.

## 5. Conclusions

In conclusion, our findings demonstrate that regional cardiac function imaging using myocardial strain analysis allows for the early differentiation of RT-induced cardiotoxicity in rats. Moreover, the use of regional cardiac function parameters measured by cardiac MRI coupled with ML analysis may be helpful for differentiating individuals who have differences in cardiac function due to a variety of factors in order to more promptly initiate intervention to avoid RIHD. Leveraging ML in this context shows great promise for improving outcomes in cancer patients by providing the cardiac effects of radiation exposure, highlighting the value of ML in advancing precision medicine in oncology.

## Figures and Tables

**Table 1:** Myocardial strain and displacement measurements in different SS.BN3 rat groups: sham, 8 weeks post-RT, and 10 weeks post-RT.

	Sham	8-wk RT (P1)	10-wk RT (P2/P3)
Ecc (%)	−14.0 ± 0.4	−14.6 ± 0.5 (0.276)	−10.9 ± 0.5 (<0.001/<0.001)
Err (%)	21.8 ± 1.6	23.1 ± 1.4 (0.334)	19.9 ± 1.8 (0.250/0.182)
Ell (%)	−16.3 ± 1.4	−15.2 ± 1.0 (0.319)	−13.6 ± 0.8 (0.066/0.127)
Rot (°)	8.0 ± 2.8	10.6 ± 3.0 (0.131)	9.6 ± 2.7 (0.178/0.332)
SAX (mm)	5.7 ± 1.3	6.5 ± 1.5 (0.011)	6.0 ± 0.2 (0.129/0.040)
LAX (mm)	5.0 ± 0.2	6.4 ± 0.5 (0.02)	7.1 ± 0.4 (0.001/0.185)

Abbreviations: Ecc, Err, Ell: circumferential, radial, longitudinal strains, respectively; Rot: tissue rotation angle; SAX: short-axis motion; LAX: long-axis motion. Values represented as mean ± standard deviation. P1, P2, and P3 show significance levels for 8 weeks post-RT vs. sham, 10 weeks post-RT vs. sham, and 10 weeks post-RT vs. 8 weeks post-RT, respectively.

## Data Availability

Restrictions apply to the datasets.

## Abbreviations

The following abbreviations are used in this manuscript:

RT	Radiation therapy
RIHD	Radiation-induced heart dysfunction
BN	Brown Norway
SS	Salt-sensitive
LV	Left ventricular
EF	Ejection fraction
MRI	Magnetic resonance imaging
HF	Heart failure
ML	Machine learning
IACUC	Institutional animal care and use committee
LAX	Long-axis
SAX	Short-axis
FLASH	Fast low angle short
TR	Repetition time
TE	Echo time
FOV	Field of view
Ecc	Circumferential strain
Err	Radial strain
Ell	Longitudinal strain
AHA	American Heart Association
RFE	Recursive feature elimination
LR	Logistic regression
SVM	Support vector machine
RF	Random forest
GB	Gradient boosting
NN	Neural networks
ROC	Receiver operating characteristic
ED	End-diastolic
ES	End-systolic
